# Markers of preparatory attention predict visual short-term memory performance

**DOI:** 10.1016/j.neuropsychologia.2011.02.016

**Published:** 2011-05

**Authors:** Alexandra M. Murray, Anna C. Nobre, Mark G. Stokes

**Affiliations:** Brain and Cognition Laboratory, Department of Experimental Psychology, University of Oxford, South Parks Road OX1 3UD, United Kingdom

**Keywords:** Visual short term memory, Attention, Precision, EDAN, ADAN, CDA

## Abstract

Visual short-term memory (VSTM) is limited in capacity. Therefore, it is important to encode only visual information that is most likely to be relevant to behaviour. Here we asked which aspects of selective biasing of VSTM encoding predict subsequent memory-based performance. We measured EEG during a selective VSTM encoding task, in which we varied parametrically the memory load and the precision of recall required to compare a remembered item to a subsequent probe item. On half the trials, a spatial cue indicated that participants only needed to encode items from one hemifield. We observed a typical sequence of markers of anticipatory spatial attention: early attention directing negativity (EDAN), anterior attention directing negativity (ADAN), late directing attention positivity (LDAP); as well as of VSTM maintenance: contralateral delay activity (CDA). We found that individual differences in preparatory brain activity (EDAN/ADAN) predicted cue-related changes in recall accuracy, indexed by memory-probe discrimination sensitivity (*d*′). Importantly, our parametric manipulation of memory-probe similarity also allowed us to model the behavioural data for each participant, providing estimates for the quality of the memory representation and the probability that an item could be retrieved. We found that selective encoding primarily increased the probability of accurate memory recall; that ERP markers of preparatory attention predicted the cue-related changes in recall probability.

## Research highlights

► Preparatory attention increases the probability that cued items enter VSTM. ► Markers of anticipatory attention were evident prior to VSTM encoding. ► ERP markers of anticipatory attention predicted attention-related increases in recall probability. ► There was no evidence that preparatory attention modulates the precision of VSTM encoding.

## 1 Introduction

The ability to hold in mind images of objects that are no longer physically present is critical for many higher-level cognitive processes. This type of ‘visual short-term memory’ (VSTM), however, is severely limited in its capacity (Phillips, 1974), and therefore only the most behaviourally relevant information should be encoded and maintained. Consequently, mechanisms of attention are likely to be important for selective encoding of behaviourally relevant information (Bundesen, 1990; Schmidt, Vogel, Woodman, & Luck, 2002). Extensive research demonstrates how preparatory attention directed to task-relevant attributes can facilitate sensory processing (Hopfinger, Buonocore, & Mangun, 2000; Luck, Chelazzi, Hillyard, & Desimone, 1997; Nobre, Sebestyen, & Miniussi, 2000; Posner, 1980). In this experiment, we explore whether and how preparatory attention may also optimise VSTM.

Surprisingly little empirical research has focused on the attentional gating into VSTM and the consequences on subsequent performance. Behavioural evidence demonstrates how a preparatory attention cue biases VSTM encoding in favour of cued items (Schmidt et al., 2002), however, previous neural studies have focused on the attentional modulations of perceptual processing in VSTM tasks rather than the preparatory state per se. For example, in a series of ERP experiments, Gazzaley and colleagues have shown that attention directed to specific memory items in a VSTM task modulates neural processing of the cued memory item (for a review see Gazzaley, this issue). Recently, Rutman, Clapp, Chadick, and Gazzaley (2010), further demonstrated that the magnitude of attention-related modulation of activity elicited by the memory item predicts VSTM performance. However, these studies did not examine the role of preparatory neural activity. Studies with a spatial manipulation of attention can, in principle, be used to examine specific anticipatory states (e.g. left vs. right). Despite this, previous studies of spatially selective VSTM do not typically report cue-related activity (Vogel & Machizawa, 2004; Vogel, McCollough, & Machizawa, 2005) or use it to predict behaviour (Griffin & Nobre, 2003). In this study, we directly examine the effect that preparatory spatial attention has on VSTM encoding and performance. Moreover, we also include manipulations of load and precision to examine more specifically which aspects of VSTM representations are facilitated by attention.

In order to investigate the influence of preparatory attention on neural markers of VSTM maintenance and subsequent behavioural performance, we relied on lateralised ERP signatures of anticipatory attention and of VSTM maintenance. The early direction attention negativity (EDAN) and the anterior attention directing negativity (ADAN) have been linked to the processing of directional cues and initiation of anticipatory shifts in spatial attention (Harter, Mille, Price, LaLonde, & Keyes, 1989; Jongen, Smulders, & Van Der Heiden, 2007; Nobre et al., 2000). Recently, Praamstra and Kourtis (2010) have suggested that the EDAN reflects visual processing in occipital cortex which precedes activations in fronto-parietal cortices manifested by the ADAN. The late directing attention positivity (LDAP; Harter et al., 1989; Yamaguchi, Tsuchiya, & Kobayashi, 1994), is a late and tonic posterior marker, thought to reflect an enhancement of visual cortical excitability in anticipation of upcoming stimuli (e.g. Jongen et al., 2007).

The contralateral delay activity (CDA) (Vogel & Machizawa, 2004; Vogel et al., 2005), also known as the sustained posterior contralateral negativity (SPCN) (e.g. Jolicœur, Brisson, & Robitaille, 2008), serves as a marker of selective maintenance in VSTM. This sustained negative potential over posterior electrodes contralateral to the selectively remembered items has been shown to scale with memory load until the capacity limit is reached (Vogel & Machizawa, 2004; Vogel et al., 2005). The relationship between the necessary preparatory attentional precedent to the CDA and VSTM performance, however, has been largely overlooked.

In this study, we examined the specific behavioural consequence of selective VSTM encoding, and related these to neural markers of selective attention and VSTM maintenance. Using a novel behavioural paradigm, we manipulated preparatory spatial attention and parametrically varied both the memory load and the precision of recall required to perform the task. This enabled us to separate the behavioural advantage of preparatory attention in terms of the quality versus probability with which selected items are maintained in VSTM (c.f. Bays & Husain, 2008; Zhang & Luck, 2008). We found that selective attention optimised VSTM encoding primarily by biasing the probability that task-relevant items will be represented, but not necessarily their representational precision. Critically, we also found that the neural markers for selection – EDAN and ADAN – predicted this behavioural cueing advantage.

## 2 Materials and methods

### 2.1 Participants

Sixteen right-handed volunteers with normal, or corrected-to-normal, vision were recruited for the study. One participant was excluded owing to noisy electroencephalographic activity (±2 standard deviations from the mean). The remaining 15 participants (age range = 22–33 years, *M* = 26.87, 8 female) were used for all behavioural and neural analyses. All participants provided informed written consent, and were reimbursed £17 for their time. To maintain motivation throughout the experiment, participants were informed that they would receive a small financial bonus (£3) for good behavioural performance, which all participants received. The experimental protocol received approval from the Central University Research Ethics Committee of Oxford.

### 2.2 Task design and procedure

**Fig. 1:**
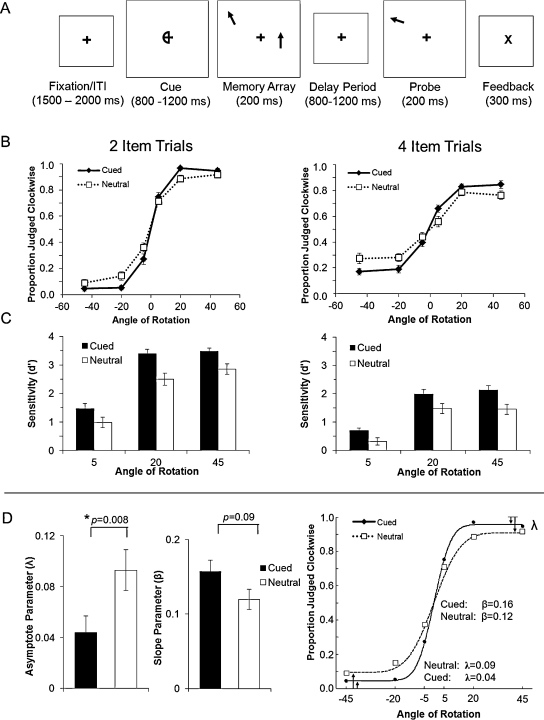
Task schematic and behavioural results. [A] On cued trials, a spatial selection cue (800–1200 ms) indicated which items to encode from a subsequent memory array. After the variable delay period (800–1200 ms), memory was assessed via a test probe. The probe appeared in the same location of a randomly selected item from the cued side of the memory array, with the important exception that it was rotated about its central axis. Participants judged whether the change was clockwise or anti-clockwise. Neutral trials were the same, except the cue provided no predictive information regarding the side from which items could be probed. [B] The proportion of probe stimuli judged clockwise as a function of the rotation angle for both cued and neutral trials and set-size (set-size 2: left; set-size 4: right). [C] Sensitivity for change discrimination is shown as a function of rotation angle (averaged across direction), cue-type and set-size. [D] The average parameter estimates across subjects for set size 2, showing asymptote (*λ*) and slope (*β*) parameters, respectively. On the right, the psychometric function generated by these average parameters is plotted against the corresponding average data points. Error bars represent ±1 s.e.m.

Adapting a task originally developed by Bays and Husain (2008), we presented arrays of arrows and then asked participants to compare their memory representation with a probe arrow which rotated clockwise or anti-clockwise. A schematic of the task is presented in Fig. 1. Each trial began with a centrally presented fixation cross (0.86° by 0.86°) for 200 ms, followed by a spatial selection cue lasting 800–1200 ms. The temporal variability in cue duration was included to reduce temporal expectation (see Griffin & Nobre, 2005). The cue was either a semi-circle presented around the fixation cross (valid cue), or a full circle (neutral cue). On validly cued trials, half the participants (randomly selected) were instructed to direct covert attention toward the hemifield indicated by the “closed” side of the cue, whereas the other half of participants were instructed to attend to the hemifield indicated by the “open” side. This manipulation ensured that cues signalling spatial attention to the left versus the right visual field were physically equated across participants. Immediately after the offset of the cue, a memory array was presented for 200 ms. The memory array consisted of two, four or eight coloured arrows, distributed across both sides of the visual display. On neutral trials, participants were instructed to encode into memory the orientation of as many arrow stimuli as possible across both visual fields. On cued trials, participants were instructed to encode only the orientation of arrows presented on the cued side. After a variable delay period (between 800 and 1200 ms), memory was probed for an item selected at random from the set of stimuli to be remembered. The probe stimulus was always an arrow presented at the same location, and with the same colour, as an item from the memory array. However, its orientation varied parametrically across trials, being rotated 5°, 20° or 45° clockwise or anti-clockwise relative to the orientation of the memoranda. Participants were instructed to indicate the direction of rotation via a key press: the right CRTL button (with right hand) for clockwise and the left CTRL button (with left hand) for anti-clockwise rotations. Visual feedback was provided after each response: stimuli for correct (centrally presented ‘£’) or incorrect (‘X’) responses were presented immediately after the response was made. Finally, after feedback, the fixation cross was presented again throughout a variable inter-trial-interval (1300–1800 ms).

Participants were given both verbal and written task instructions before the task began. The task was completed in a quiet, dimly illuminated, and electrically shielded room. Head position was stabilised using a chin rest positioned 100 cm from a computer monitor. Eye position was monitored throughout the experiment using a remote infrared eye tracker (ISCAN Inc., Woburn, USA). Participants were asked to refrain from blinking during the active portions of the task and, if necessary, to blink just after responding to the probe stimulus. Participants completed one block of twenty practice trials to become familiar with the task; followed by ten experimental blocks of 72 trials (720 trials in total). Breaks between blocks were self-paced. The order of trials with each cue type, set size and rotation change conditions was randomised across the entire experiment.

### 2.3 Stimuli

The stimuli were constructed using Presentation (Neurobehavioral Systems, Albany CA, USA) software on a trial-by-trial basis, and presented against a black background. The fixation cross and cue stimuli were light grey and subtended 0.86° by 0.86° of visual angle. The cues were formed by adding either one (cued trials) or two (neutral trials) semi-circles to the fixation cross. Memory items were arrows (subtending 1.43° from base to tip) presented at a randomly selected orientation (angles equally distributed between 0 and 2π). Memory stimuli were presented at randomised locations within 2 bounding boxes, 23.44° (vertical) by 13.67° (horizontal), centred at 6.8° to the left and right of fixation. Each arrow was separated by a minimum distance of 2.39° to reduce perceptual effects of crowding. The colour of each memory item was chosen at random, and without replacement, from a selection of easily discriminable colours (Red, Blue, Green, Cyan, Yellow, Magenta, Orange or White). Behavioural feedback, presented directly after the response, was a light grey “£” for correct, and an “X” for incorrect (in Arial, 28pt font, approximately 0.86° by 0.86°).

### 2.4 Behavioural analysis: *d*′, recall probability and precision

Behavioural analyses were conducted on estimates of sensitivity (*d*′) for change discrimination (Swets & Green, 1966). The *d*′ scores were calculated with respect to the standardised proportion of trials in which participants responded clockwise on clockwise (hits) versus anti-clockwise change trials (false alarms):

$$d^{\prime }=z(\text{hit})-z(\text{false}\;\text{alarm})$$

Behavioural data were also modelled to estimate two parameters: *λ* (asymptotic performance: the probability that items were maintained in VSTM; Zhang & Luck, 2008) and *β* (slope or precision of the memory representation; Bays & Husain, 2008) according to the following equation:

$$y=\lambda +\frac{\left(1-2\lambda \right)}{2}\cdot erfc\left(\frac{-\beta }{\sqrt{2}}(x)\right)$$

where *erfc* is the complementary Gaussian error function. These estimates were modelled using the Matlab toolbox Palamedes (Prins & Kingdom, 2009). Estimates for both slope and asymptote were unconstrained across the cue conditions and, because differences in performance between clockwise and anti-clockwise were not important in this experiment, we did not include a parameter for the threshold. Similarly, we only included the single parameter, *λ*, for both the guess and lapse to capture the performance asymptote (analogous to Zhang & Luck, 2008).

A larger value for *β* reflects a steeper slope, which we interpret as a more precise representation stored in VSTM (see Bays & Husain, 2008; Zhang & Luck, 2008). Specifically, when participants have a more precise representation, the degree of change required to make a correct response is comparatively smaller than when the representation is coarse. Estimates of *λ*, on the other hand, can be interpreted as an index of the maximum number of items that are available in VSTM at the time of the test probe (Zhang & Luck, 2008). More specifically, *λ* reflects the upper bound estimate for recall accuracy if the change discrimination were infinitely easy.

### 2.5 Electroencephalography

#### 2.5.1 EEG recording

The EEG was recorded continuously using NuAmp amplifiers (Neuroscan Inc., Albany, USA) from 40 silver/silver chloride electrodes positioned according to the 10–20 international system (AEEGS, 1991). Recordings were taken from electrodes: Fz, FCz, Cz, CPz, Pz, POz, Oz, FP1/2, F3/4, F7/8, FT7/8, T7/8, TP7/8, FC3/4, C3/4, CP3/4, P3/4, P7/8, PO3/4, PO7/8, and O1/2. Blinks and eye movements were monitored by deriving bipolar recordings from an electrode placed below the right eye and FP2 (VEOG) and from electrodes placed to the left and right of the right eye (HEOG). The electrode placed at AFz served as the ground, and the left mastoid served as the active reference. Electrical impedances were kept below 5 kΩ and activity was filtered with a low-pass filter of 300 Hz. The analogue-to-digital sampling rate of brain activity was set at 1000 Hz and data were recorded continuously for the entire experiment. Digital codes were also sent via the parallel port by the stimulus presentation computer to mark the moments of various stimuli onset for each trial.

#### 2.5.2 EEG processing

All further processing and analyses were done offline. Firstly, the EEG was re-referenced to the average of the left and right mastoids. The two bipolar electro-oculogram (EOG) signals were derived by calculating the difference between the upper and lower HEOG electrodes and between the left and right VEOG electrodes. Data were then cut into separate cue-locked and memory array-locked epochs (−200 to 1000 ms) for each trial.

Pre-processing algorithms removed epochs with excessive noise or drift (exceeding ±100 μV at any electrode) from subsequent analysis. In addition, epochs containing blinks or eye movements (exceeding ±50 μV from the EOG channels) were excluded. All epochs were then visually inspected, and any epochs with detectable smaller eye movements were removed manually. We also rejected error trials. After pre-processing, cue-related ERPs were derived by averaging cue-locked epochs according to the two cue directions. Memory-array ERPs were derived according to cue direction and memory load. We were only interested in neural activity that was lateralised with respect to the attention cue, and correspondingly, the spatial location of items in VSTM. To increase trial numbers, data from left- and right-cue trials were combined in a way that preserved the spatial positioning of the electrodes relative to the side indicated by the cue (i.e., ipsilateral and contralateral). To ensure quality of the ERP data, we set a minimum criterion of 25 epochs per condition. Because of low performance accuracy, combined with the usual EEG artefacts, the experimental conditions including 8-item arrays failed to reach this criterion in a large proportion of the participants and could not be included in the analyses. The ERP analysis, therefore, focused on the conditions with 2- and 4-item arrays only.

Data were then imported into Matlab (MathWorks, Natick, USA) and baseline-corrected. The 200 ms period before cue onset was used as the baseline for both cue-locked and memory-array-locked ERPs. This is important to eliminate the possibility of any lateralised activity occurring as a result of anticipatory spatial attention before appearance of the memory array (e.g. tonic effects related to the cue-related LDAP) potentially contaminating the lateralised VSTM maintenance-related activity (CDA). This is especially important in the current experiment because the LDAP (related to lateralisation of pre-stimulus visual excitability) and the CDA (related to lateralisation of VSTM maintenance) are modulated in opposite directions (enhanced contralateral positivity and negativity, respectively). Using the time period immediately before the onset of the memory array as a baseline would conflate sustained measures of lateralisation related to anticipatory spatial attention and VSTM maintenance, and thereby exaggerate the magnitude of delay activity reflected in the CDA.

#### 2.5.3 ERP analysis

To investigate the neural underpinnings of cue-related processing, and their relationship to behaviour, we quantified the mean voltage amplitudes of lateralised ERPs previously associated with preparatory spatial biases of attention (EDAN, ADAN, LDAP). Repeated-measures analyses of variance (ANOVAs) were used to compare neural activity across the conditions of interest. EDAN was quantified as the mean voltage difference between contralateral and ipsilateral sites over P7/8 and O1/2 at 250–400 ms after cue onset (Jongen et al., 2007; Praamstra & Kourtis, 2010); ADAN was quantified as the mean amplitudes in the anterior electrodes sets (FC3/4, C3/4) averaged over the period 350–500 ms after cue onset (Jongen et al., 2007); LDAP was quantified as the mean amplitude over electrodes (PO3/4) averaged over 550–800 ms after cue onset (McDonald & Green, 2008; Van Velzen & Eimer, 2003). The CDA was quantified using mean amplitudes (between 450 and 800 ms post memory array onset) at PO7/8 (Vogel & Machizawa, 2004).

Where appropriate, the Greenhouse-Geisser correction was used to control for effects of non-sphericity. We also tested how the magnitude of anticipatory markers of spatial attention (EDAN, ADAN and LDAP) and maintenance activity (CDA) could predict behavioural cueing effects. Measurements of *d*′ were averaged across set sizes. Model parameter estimates, however, were only stable for set size 2 so these correlation analyses focused on data from the smaller set size. The functional link between these ERPs and behavioural measures was assessed using split-half independent *t*-tests and Pearson's correlations (one-tailed).

## 3 Results

### 3.1 Behavioural results

The behavioural results are shown in Fig. 1. Note that we have named each condition according to the total number of items that were presented despite the fact that participants were required to only maintain half the number of items on cued trials. As expected, accuracy decreased for trials with smaller changes in rotation and for the larger set sizes, and performance was better for cued, relative to neutral, trials. Statistical effects were assessed via a 2 (cue type: valid and neutral) × 2 (set size: 2 or 4 items) × 3 (angle of rotation: 5°, 20°, 45°) repeated-measures ANOVA. Sensitivity (*d*′) was significantly higher in trials with spatial cues compared to neutral cues (cued > neutral: *F*(1,14) = 27.61, *p* < 0.001). There were also significant main effects for set-size (2 item > 4 items: *F*(1,14) = 145.64, *p* < 0.001) and change magnitude (linear trend, *F*(1,14) = 208.64, *p* < 0.001). Finally, there was a significant interaction between set size and rotation change (*F*(2,28) = 8.22, *p* = 0.002), due to a significantly smaller difference between set sizes at the small (5°) compared to the medium (20°; *p* = 0.014), and large rotation change (45°; *p* = 0.015). No other terms reached significance (*p*s > 0.3).

### 3.2 Modelling results

Model parameters for slope (*β*) and asymptote (*λ*) were estimated for all set sizes, however, only data from set size 2 yielded stable results. Consistent with the results described above for *d*′, a paired-samples *t*-test revealed that there were significantly lower asymptote estimates for cued compared to neutral trials (*t*(14) = 3.09, *p* = 0.008, see Fig. 1). This effect suggests that preparatory attention can increase the probability that items are maintained in VSTM. There was also a trend for higher slope estimates for cued relative to neutral trials (trend: *t*(14) = 1.81, *p* = 0.092, see Fig. 1). Together, these results are consistent with evidence that attentional orienting effectively reduces the encoding set size, which in turn increases the probability that items are maintained in VSTM and may also increase the precision with which they are maintained (Bays & Husain, 2008), at least when the total number of items is relatively low (Zhang & Luck, 2008).

### 3.3 Cue-related ERPs

**Fig. 2:**
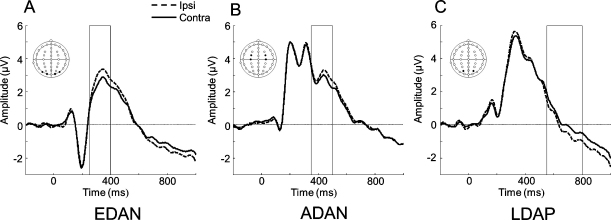
Grand-averaged ERPs time-locked to the cue. Solid lines indicate activity from contralateral sites whereas dashed lines indicate activity from ipsilateral sites. Boxes highlight the time period of interest, during which ipsilateral and contralateral activity differed significantly. [A] EDAN – activity for ipsilateral and contralateral sites for electrodes P7/8 and O1/2 from 250 to 400 ms, [B] ADAN – activity for ipsilateral and contralateral sites for electrodes FC3/4, C3/4 from 350 to 500 ms, [C] LDAP – activity for ipsilateral and contralateral electrode sites for PO3/4 from 550 to 800 ms.

As shown in Fig. 2, we observed lateralised markers of anticipatory attention (EDAN, ADAN and LDAP) during the cue period, before the memory array was presented. To test for the presence of these markers, repeated-measures ANOVAs tested for the effect of relative hemisphere (ipsi vs. contra) as well as electrode pair where relevant. Significant differences in mean voltages were observed in each case: EDAN (contra < ipsi: *F*(1,14) = 5.89, *p* = 0.029); ADAN (contra < ipsi: *F*(1,14) = 6.57, *p* = 0.023); LDAP (contra > ipsi, *F*(1,14) = 5.01, *p* = 0.021); No other significant terms were observed (*ps* > 0.17).

### 3.4 ERP memory maintenance results

**Fig. 3:**
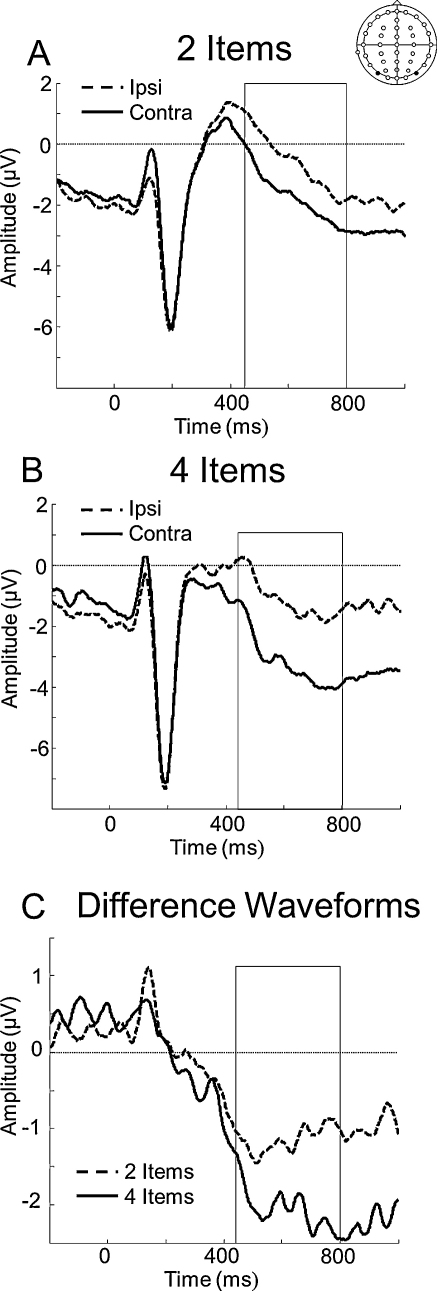
Grand-averaged ERPs time-locked to the memory array. Solid lines indicate activity from contralateral sites whereas dashed lines indicate activity from ipsilateral sites. Boxes highlight the time period of interest, during which potentials differed significantly between ipsilateral and contralateral electrodes. [A] CDA – ipsilateral (dashed line) and contralateral (solid line) activity from PO7/8 for 2 item trials from 450 to 800 ms, [B] CDA – ipsilateral (dashed line) and contralateral (solid line) activity from PO7/8 for 4 item trials from 450 to 800 ms, [C] The difference waveform (contralateral minus ipsilateral activity) for the 2-item (dashed line) and 4-item (solid line) trials from 450 to 800 ms. Note that the ERPs and the subtraction waveforms do not begin at 0 μV because a pre-cue baseline was used (see Section 2.5.2).

The ERP elicited by the memory array can be seen in Fig. 3. Mean amplitudes at PO7/8 (450–800 ms) were submitted to a two-way repeated measures ANOVA, with factors for set size (2 or 4 items) and electrode side (ipsilateral or contralateral), which confirmed a greater negativity for contralateral, relative to ipsilateral, electrodes (*F*(1,14) = 16.74, *p* = 0.001). Importantly, we also observed a significant set size by hemisphere interaction (*F*(1,14) = 11.52, *p* = 0.004), demonstrating a selective negativity increase for 4- relative to 2-items at contralateral (*p* = 0.05), but not ipsilateral electrodes (*p* = 0.37).

### 3.5 Electrophysiological predictors of behaviour

Next, we explored the relationship between the electrophysiological markers of preparatory attention reported above, and VSTM performance for each participant. First, we found that individual differences in ADAN magnitude predicted individual differences in *d*′ cueing effect (difference in *d*′ for cued and neutral trials; *r*(13) = −0.50, *p* = 0.03). A similar trend was found for EDAN (*p* = 0.09), but not for LDAP (*p* = 0.37). Splitting the participants into those who showed a large or small advantage in *d*′ for cued as opposed to neutral trials revealed a significant difference in EDAN magnitude (*t*(13) = −3.49, *p* = 0.002) and a trend for ADAN (*t*(13) = −1.68, *p* = 0.059).

**Fig. 4:**
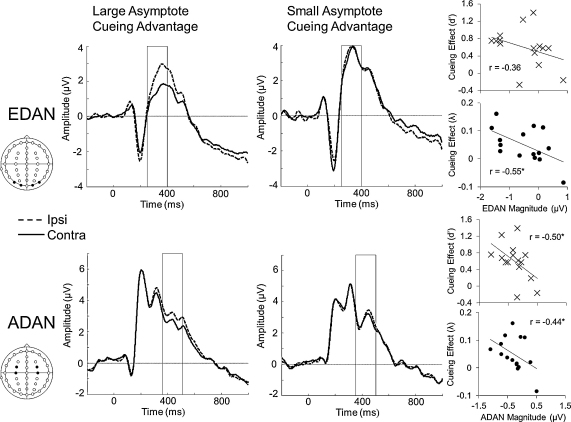
Neural predictions of the advantage in asymptote. The magnitude of the EDAN (top row) and ADAN (bottom row) effect split according to participants with relatively large (left panels) or small (middle plots) difference in asymptote (neutral-cued). Boxes indicate the time period of interest: 250–400 ms for EDAN and 350–500 ms for ADAN. During these time periods, there was a significant difference between participants who show a large compared to a small cueing advantage in asymptote. The right-hand panels show the correlations between cueing advantage for *d*′ and for asymptote and EDAN magnitude (top two panels, respectively) and ADAN magnitude (bottom two panels, respectively). * Indicates a significant correlation (*p* < 0.05).

We observed complementary effects comparing the difference between neutral and valid asymptote estimates and the magnitude of our preparatory attention cueing effects. The difference in asymptote estimates between cued and neutral trials at the smallest set size was predicted by EDAN (*r*(13) = −0.55, *p* = 0.016, see Fig. 4, top row), and ADAN (*r*(13) = −0.44, *p* = 0.049, see Fig. 4, bottom row). Independent samples *t*-tests performed on EDAN and ADAN magnitudes confirmed this result. EDAN (*t*(13) = −2.19, *p* = 0.024, see Fig. 3, top row) and ADAN (*t*(13) = −1.68, *p* = 0.029, Fig. 4, bottom row) magnitudes were greater (in the negative direction) for those who showed a large compared to a small cueing difference in asymptote. Once again, LDAP was not predictive of this behavioural cueing effect (correlation; *p* = 0.12; split-half; *p* = 0.947).

In contrast to the brain–behaviour relationships that we observed for the cue-related behavioural advantage measured in *d*′ and the asymptote of the psychometric function, we did not observe any similar relationships for the slope estimates. The difference in slope-parameter estimates between neutral and valid trials for the lowest set size was not predicted by EDAN (*p* = 0.18), ADAN (*p* = 0.09), or LDAP (*p* = 0.90).

**Fig. 5:**
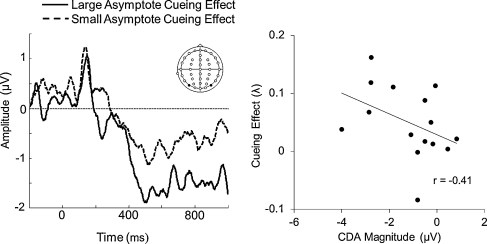
The relationship between CDA magnitude and the cueing effect in asymptote. The left-hand panel shows the difference waveform between contralateral and ipsilateral sides of space for 2-item trials split according to whether the participants had a large (solid line) or small (dashed line) difference in asymptote (neutral - cued). The right-hand panel shows the relationship between maintenance activity and cueing advantage for asymptote estimates. Note these effects are not significant (during the CDA time period: 450–800 ms) but are presented for completeness.

Finally, we also explored whether maintenance-related delay activity correlated with measures of performance in our task. Similar to the effects with EDAN and ADAN, CDA magnitude also tended to be related to an increase in the cueing advantage in asymptote. At the smallest set size, we saw a trend suggesting that the differences in asymptote estimates between neutral and cued trials were related to differences in CDA (trend: *r*(13) = −0.41, *p* = 0.067, see Fig. 5, right panel; for all other behavioural cueing measures, *ps* > 0.1). However, an independent samples *t*-test performed on the median split of CDA amplitude by cueing asymptote differences was not significant (*t*(13) = −1.14, *p* = 0.14).

## 4 Discussion

We recorded EEG activity while participants performed a VSTM task that manipulated the spatial distribution of attention at encoding, the memory load, and the relative difficulty of the change discrimination at the memory probe. The parametric manipulation of change magnitude allowed us to model separately the number of items held in VSTM (Zhang & Luck, 2008, 2009), and the average precision of these memoranda (Bays & Husain, 2008). Selective encoding increased the number of task-relevant items that were available for recall. In contrast, there was only a trend for cue-related changes in precision. Importantly, the benefit in item-recall probability and *d*′ for cued compared to neutral trials was predicted by preparatory measures of attention, including EDAN and ADAN. In contrast, we found no relationship between preparatory brain activity and effects of cueing on precision.

The implications of these results are important for a number of reasons. Firstly, at the behavioural level, we demonstrate how preparatory orienting of spatial attention can influence the allocation of VSTM resources to favour task-relevant items. By modelling the slope and asymptote of behavioural psychometric functions for change discrimination between the memory array and test probe, we found that attention primarily optimises the number of task-relevant items encoded into VSTM. There was only a trend for a cue-related increase in precision. Previously, Bays and Husain (2008) showed that increasing the number of items stored in VSTM reduces the precision with which each item is represented. Presumably, if participants are able to use the cue to encode selectively only the task-relevant subset of items in the memory array, then more resources can be allocated to each of these items, increasing the precision with which each item is represented. The trend we observed for a cue-related increase in precision at the smallest set size is, therefore, consistent with a flexible resource model of VSTM capacity (Bays & Husain, 2008). However, our results are also compatible with less flexible models of VSTM capacity which allow for some flexibility for allocating resources when the total number of items is below capacity limit (Zhang & Luck, 2008).

In this task, we also observed a sequence of neural markers for attentional orienting during the cue period. These potentials linked to preparatory attention have not typically been considered in selective VSTM tasks, but instead have been investigated in the context of tasks requiring perceptual judgements of incoming stimuli (e.g. Jongen et al., 2007; Nobre et al., 2000; Talsma, Slagter, Nieuwenhuis, Hage, & Kok, 2005). In these cases, ERP markers of preparatory attention have been associated with a modulation of the perceptual processing of cued stimuli (Hopf & Mangun, 2000; Jongen et al., 2007; Nobre et al., 2000; Talsma et al., 2005). Our results extend these previous investigations by demonstrating that similar neural mechanisms of preparatory attentional selection can be engaged and are beneficial in preparing for selective VSTM encoding. Critically, we demonstrated a functional link between the neural mechanisms of selection signalled by EDAN and ADAN and subsequent VSTM performance, and we were also able to shed further light on the functional mechanisms for these performance benefits. Participants who showed larger markers of preparatory attention (EDAN and ADAN) showed a greater cue-related advantage for recall probability (asymptote) and *d*′. This functional link suggests that the selection processes signalled by EDAN and ADAN increase the probability that only task-relevant items will be encoded. Our findings are compatible with a related study that also suggests that ADAN is associated with filtering demands and the selection of task-relevant items (Seiss, Driver, & Eimer, 2009). In contrast, the preparatory mechanisms elicited by the pre-cue did not correlate with changes in precision, suggesting that attention facilitates memory performance by biasing the quantity, rather than quality, of cued items that are encoded into VSTM.

As a corollary, the relationship between EDAN and cued behavioural advantages (for asymptote and *d*′) also sheds light on the neural underpinnings of this potential. The functional significance of EDAN as a marker of spatial attention has been questioned (see Van Velzen & Eimer, 2003). Although explanations strictly based on the EDAN being linked to physical properties of cueing stimuli had been ruled out (Nobre et al., 2000), it remained possible that the EDAN reflected merely the ‘spatial selection’ of the aspects of the cue that were relevant to extracting its meaning. In this interpretation the EDAN is similar to the posterior lateralised N2PC potential elicited when a target is detected within a visual search array (e.g. Kuo, Rao, Lepsien, & Nobre, 2009). Explanations of this type do not readily apply to our experiment, since we used identical cueing stimuli whose prominent side was factorially crossed with the cued side. More importantly, the functional relevance of this potential to spatial selection of items for subsequent encoding was clearly evident in the strong relationship between the magnitude of this potential and subsequent behavioural performance. In this respect, our results agree with Praamstra and Kourtis (2010) who suggest that the EDAN is the posterior precedent to the ADAN. More generally, the results demonstrate the functional significance of early markers of attentional orienting.

Interestingly, we did not observe any link between the later marker of preparatory changes in visual excitability (LDAP) and subsequent VSTM performance. The stimuli in the memory array were supra-threshold and easy to see, therefore, the main limits of performance were related to the number of items available for encoding and the precision with which they had to be remembered. Consequently, heightened visual excitability may not have been advantageous for accurate performance in our task. Rather, spatial selection of the relevant items to encode, reflected in the EDAN and ADAN, was presumably more important for performance than enhancing the initial visibility of items in the memory array (see Seiss et al., 2009).

In addition to the ERP signatures of preparatory attention, we also identified load-dependant CDA. Typically thought to index the number of items being maintained in VSTM (Jolicœur et al., 2008; Vogel & Machizawa, 2004; Vogel et al., 2005), CDA has also been linked to individual capacity limits (Vogel & Machizawa, 2004) and selectivity efficiency (Vogel et al., 2005). These results are consistent with load-dependent delay activity observed in this task. The trend we observed relating CDA magnitude and the cueing difference in recall probability is also consistent with previous evidence linking maintenance activity with the ability to select task relevant items (Vogel et al., 2005).

The results of the current experiment provide important new insights into the neural mechanisms that mediate attentional selection for VSTM encoding. We found both behavioural and neural evidence that attention affects the probability that an item enters VSTM, and is successfully maintained. We suggest that these mechanisms of attention critically shape the contents of VSTM to represent only the most behaviourally relevant visual information. Recent studies are beginning to show that VSTM performance can also be enhanced by cues presented after encoding, during the VSTM maintenance period (Griffin & Nobre, 2003; Landman, Spekreijsea, & Lamme, 2003). In future studies, it will be interesting to investigate whether, at this later stage, attention can continue to influence the selection of items predicted to be relevant for behaviour or whether alternative mechanisms are employed, such as the improvement of precision with which the encoded items are represented.

## Funding

This work was supported by the Wellcome Trust [WT082791MA]. In addition, AM was supported by a Commonwealth Scholarship and MS was supported by St John's College.
