# Supplementary material for: Markers of preparatory attention predict visual short-term memory performance
Source: Neuropsychologia. 2011 May;49(6):1458–65. doi: 10.1016/j.neuropsychologia.2011.02.016 (PMC3318119; doi:10.1016/j.neuropsychologia.2011.02.016)
Supplement: Supplementary file 1 [file mmc1.doc]

Supplementary Material

Table 1

*Mean proportion correct and standard deviation for each angle of rotation for the set size 8 condition*

Mean *(SD)*

| Cue Type | -5 | 5 | -20 | 20 | -45 | 45 |
| --- | --- | --- | --- | --- | --- | --- |
| Valid | 0.55 *(0.14)* | 0.58 (0.11) | 0.57 *(0.13)* | 0.72 *(0.09)* | 0.61 *(0.15)* | 0.69 *(0.10)* |
| Neutral | 0.54 *(0.16)* | 0.58 *(0.15)* | 0.60 *(0.18)* | 0.65 *(0.13)* | 0.58 *(0.11)* | 0.67 *(0.13)* |

Table 2

*Mean sensitivity (d’) and standard deviation for each angle of rotation for the set size 8 condition*

Mean *(SD)*

| Cue Type | 5 | 20 | 45 |
| --- | --- | --- | --- |
| Valid | 0.34 *(0.41)* | 0.80 *(0.43)* | 0.84 *(0.55)* |
| Neutral | 0.34 *(0.53)* | 0.70 *(0.53)* | 0.65 *(0.50)* |
