# Supplementary material for: Markers of preparatory attention predict visual short-term memory performance
Source: Neuropsychologia. 2011 May;49(6):1458–65. doi: 10.1016/j.neuropsychologia.2011.02.016 (PMC3318119; doi:10.1016/j.neuropsychologia.2011.02.016)
Supplement: Supplementary file 3 [file mmc3.doc]

Mean *(SD)*

| Cue Type | 5 | 20 | 45 |
| --- | --- | --- | --- |
| Valid | 0.34 *(0.41)* | 0.80 *(0.43)* | 0.84 *(0.55)* |
| Neutral | 0.34 *(0.53)* | 0.70 *(0.53)* | 0.65 *(0.50)* |
